# Postal Rates

**Published:** 1912-02

**Authors:** 


### Postal Rates.

We have received several communications from publishers asking our coöperation to oppose certain projected changes in postage, and to favor other changes not projected but highly desirable. We have hesitated to respond, on account of the policy of limiting matter as closely as possible to strictly medical topics. However, this is a matter that concerns us all and we therefore, present the following views for what they are worth.

1. The Post Office should be self-supporting but not a source of revenue, beyond a small margin of safety.

2. At present, greater efficiency of service is more needed than a lowering of rates.

3. The Post Office should monopolize, so far as practicable, the transmission of personal messages, printed matter of the nature of news, literature, etc., and packages, of all sorts up to the maximum practicable limit of size and weight. The inclusion of messages requiring speed and the reduction of prices on packages is more important to the people than a slight reduction of rates on matter at present handled by post. Yes, call us a paternalist, socialist or anything else you please, we mean government handling of the telephone, telegraph and express. Increased facilities for local collection, sorting and delivery should also be provided, at least to the point that a message and answer may be obtained with certainty in any one day, in the same town.

4. There should be an extension, rather than a reduction of postal facilities on Sunday, for exactly the same reasons that street cars should run on Sunday.

5. We do not believe in reducing letter postage generally. There are too many persons who ought to be plowing, laying bricks, selling underwear, running typewriters and washing dishes, but who are frittering away their time trying to be authors. These persons should not be encouraged by making the mailing of manuscripts cheaper. Another reason is that it is worth something to be able to deposit circulars promptly in the waste paper basket.

6. Against our own interests, we believe that publishers should not be unduly favored. Too many trees are being ground up to make paper and there is too much eye strain and waste of time in reading the great mass of "literature" with which the country is flooded. Publishers should pay what the transmission of their periodicals cost, with due regard to the bulk of business afforded, and the fact that not so much haste is required as in the handling of letters.

7. We do not regard it as feasible to distinguish between reading matter and advertising in charging postage rates on magazines, etc., and while we believe that there should be a discrimination between magazines issued solely or mainly as advertisements and bona fide periodicals, we think the regulations should be altered so as to allow free sampling. Rather should each magazine be judged on its own merits.

8. We hold that it is manifestly unfair to charge more for delivery in the city of publication than 3,000 miles away.

9. Foreign postage should be reduced to a single uniform price.

